# Excess mortality for men and women above age 70 according to level of care during the first wave of COVID-19 pandemic in Sweden: A population-based study

**DOI:** 10.1016/j.lanepe.2021.100072

**Published:** 2021-03-17

**Authors:** K. Modig, M. Lambe, A. Ahlbom, M. Ebeling

**Affiliations:** a Unit of Epidemiology, Institute of Environmental Medicine, Karolinska Institutet, Stockholm, Sweden; b Institute of Epidemiology and Biostatistics, Karolinska Institutet, Stockholm, Sweden; c Regional Cancer Centre, University Hospital, Uppsala, Sweden; d Max Planck Institute for Demographic Research, Rostock, Germany

## Abstract

**Background:**

Both age and comorbidity are established risk factors for death among those infected with COVID-19. Because they often co-exist, it is difficult to assess if age is a risk factor on its own.

**Methods:**

We used administrative register data of the total Swedish population from 01/2015 until 07/2020. We stratified the population aged 70+ into three groups according to level of care (in care homes, with home care, and in independent living). Within these groups, we explored the level of excess mortality in 2020 by estimating expected mortality with Poisson regression and compared it to observed levels. We investigated if excess mortality has been of the same magnitude in the three groups, and if age constitutes a risk factor for death during the pandemic regardless of level of care.

**Findings:**

Individuals living in care homes experienced the highest excess mortality (75- >100% in April, 25–50% in May, 0–25% in June, depending on age). Individuals with home care showed the second highest magnitude (30–60% in April, 15–40% in May, 0–25% in June), while individuals in independent living experienced excess primarily at the highest ages (5–50% in April, 5–50% in May, 0–25% in June). Although mortality rates increased, the age-pattern of mortality during the pandemic resembled the age-pattern observed in previous years.

**Interpretation:**

We found stepwise elevated excess mortality by level of care during the first wave of the COVID-19 pandemic in Sweden, suggesting that level of frailty or comorbidities plays a more important role than age for COVID-19 associated deaths. Part of our findings are likely attributable to differences in exposure to the virus between individuals receiving formal care and those living independently, and not only different case fatality between the groups. Although age is a strong predictor for mortality, the relative effect of age on mortality was no different during the pandemic than before. We believe this is an important contribution to the discussion of the pandemic, its consequences, and which groups need the most protection.

**Funding:**

This study was funded by the Swedish Research Council for Health, Working Life and Welfare (FORTE: grant 2016-07115).

**Research in context**

**Evidence before this study**

Old age and comorbidity are both known to increase the risk of dying from COVID-19. This has been observed in the general population and COVID-19 patients. It could, however, be hypothesized that old age has a less pronounced role in risk of death from COVID-19 in healthier subgroups of the population than among frail or ill individuals. Furthermore, the role of individuals’ living arrangement, whether in care homes or independent living, for death from COVID-19 is also of importance in the context of evaluating policies aimed at protecting vulnerable groups. There is a lack of studies exploring the role of health status, living arrangement, and age in the total population considering the size of each subgroup.

**Added value of this study**

To the best of our knowledge, this study is the first to estimate age-specific risk of death during the pandemic in population groups stratified by level of care, which, in this setting, is also a rough proxy for level of frailty and underlying health status. Age-specific excess mortality during the first wave of the COVID-19 pandemic in Sweden is presented by three levels of care in individuals aged 70 years and older. Furthermore, this study shows the extent to which excess mortality levels have clearly differed between the three levels of care at a given age. Care homes have been at the center of attention in the discussions of how successful pandemic polices have been. It is clear from our results that the policy of protecting the old and vulnerable was not very successful during the first wave of the pandemic in Sweden.

**Implications of all the available evidence**

Age has a clear impact on the risk of death, and the risk of death increases with every passing chronological year. However, during the pandemic, the relative effect of age on mortality has not been different than before, as indicated by the almost parallel upward shift of death rates. Our findings suggest that level of care, and perhaps health status, play a more important role than age for COVID-19 associated deaths. Therefore, this suggests that health status should perhaps be given more attention when protecting vulnerable groups than age alone. Part of the findings are likely attributed to differences in exposure to the virus between individuals receiving formal care and those living independently.

Alt-text: Unlabelled box

## 1 Background

Old age, as well as poor health, have been identified as major risk factors for dying with COVID-19 ([1, 2]). Because these risk factors often co-exist, it is difficult to distinguish their respective role for adverse outcomes. It is, however, important to understand the relative roles of age and health status for the risk of dying with COVID-19, to identify individuals at risk. While age has been communicated as the primary risk factor for mortality from COVID-19, alternative approaches to using chronological age as the sole criterion for allocating medical resources have been discussed [3].

A review and meta-analysis found a weak influence of age on COVID-19 disease severity when adjusting for important age-dependent risk factors [4]. However, few studies met the inclusion criteria. A Swedish study including patients with and without COVID-19 admitted to a geriatric clinic found that clinical frailty was a more important predictor for death with COVID-19 than age [5]. In addition, a US-based study using information from electronic medical records found that age had only a small effect on COVID-19 mortality when comorbidities were considered [6]. However, the existing literature has failed to address two important questions regarding age and COVID-19 mortality. First, what the age trajectory of mortality during the pandemic looks like, in comparison to previous years, and if this differs by groups with different health status. Often age is adjusted for but how death risk increases with age is rarely presented. Age is among the strongest risk factors for mortality, even in the absence of a pandemic, with an exponential increase in death risk with age [7]. The question is, has age had a more pronounced role on mortality during the pandemic, and, if so, has this age effect been different in groups with different health status and care need? A reasonable strategy to assess the risk of dying from COVID-19 in different groups is to stratify the population accordingly and estimate death rates within each subgroup separately.

Second, until recently, most studies have been based on COVID-19 cases only, and most often on patients with severe COVID-19 who were admitted to the hospital ([8, 9]). While this can be informative in a clinical setting, it precludes conclusions about the relative role of age and health status during the pandemic in the total population. Relative risks of certain risk factors in relation to others are valid only among the infected individuals under study or generalized to subgroups of other infected individuals. They are, however, not valid as risk estimates for the same factors within the total population. It could be hypothesized that old age or comorbidity level have a less pronounced role in COVID-19 death among healthier subgroups of the population than among frail or ill subgroups, where a strong selection has already taken place.

Excess mortality has been reported in many studies during the pandemic [10], [11], [12], and also sometimes for in care- or nursing home populations [13]. Yet it is not entirely clear how excess mortality compares with expected mortality levels in different age groups, as well as for individuals in different settings, such as with and without home care.

In Sweden, elderly care is dominated by the principle of “age-in-place”, meaning that old individuals continue to live at home for as long as possible [14]. With time, they are offered publicly organized and funded home care to manage everyday life. This care consists of assistance with everyday activities, such as cleaning, groceries, and cooking, and personal care, such as showering and going to bed. A smaller proportion also receives home health care. If individuals lose the ability to live at home even with assistance, they are offered the opportunity to live in a care home. Once individuals move to a care home, they are generally frail and suffer from a high burden of comorbidity, which is reflected by the statistic that, as of 2018, older adults spent only a median 2 years in a care home before dying [15]. Here we distinguish three groups in the total population according to their level of care, which may be considered a proxy of health status. These groups were individuals aged 70 years and older i) in independent living, ii) with home care, and iii) in care homes.

The aim of the present study was two-fold. First, to examine if age-specific excess mortality during the first wave of the COVID-19 pandemic in Sweden, between January and July 2020, differed by level of care in individuals 70 years and older. Second, to assess how the increase in death risk with age differed across levels of care.

## 2 Material and methods

### 2.1 Data

Our analyses were based on data from the Swedish Cause of Death Register [16] and the Register of Municipal Care for elderly and individuals with disabilities. In accordance with the Social Services Act, both are administered by the National Board of Health and Welfare and updated monthly in this study. We analyzed monthly all-cause death counts for Sweden between January 2015 and July 2020, stratified by month, sex, age, and care status. The data was structured into five age groups, ranging from 70–74 to 90+. The three distinct groups for care status were: individuals living at home without assistance (“independent living”), individuals living at home receiving home care (“home care”), and individuals residing in care homes (“care home”). Information about the number of individuals in each group and the number of deaths that occurred in the three groups was obtained at the beginning and end of each month, respectively.

Currently available information makes it difficult to assess with certainty who has died from COVID-19. For instance, COVID-19 deaths are inherently uncertain because their classification depends on intensity of testing, principles for assigning cause of death, and whether all or only hospitalized deaths are considered [17]. We therefore chose to assess the excess of total mortality (all causes) in 2020 compared to previous years. However, in secondary analyses, we compared total excess mortality and COVID-19 deaths, the latter defined as death within 30 days of confirmed COVID-19 by PCR-test.

### 2.2 Estimating observed mortality in 2020

Monthly death counts and person years at risk for each month and care group by age and sex served as input for the estimation of the observed mortality rates in 2020. Person years were estimated by averaging the respective start-month and end-month population and multiplying it by the proportion of days in a given month among the total number of days in the respective year.

### 2.3 Estimating expected mortality in 2020 in the absence of COVID-19

Expected mortality in 2020 for the three care groups is based on the mortality trend in the previous 5 years. We estimated expected mortality in 2020 using a Poisson GLM, which was the preferred choice in a comparison with a negative binomial GLM. We estimated separate models for each age group, care status group, and sex. Our model specification followed a Serfling-like regression model, which has frequently been used for the estimation of baseline mortality in seasonal settings [18]. Each model consisted of a time trend component that we modeled using a natural cubic spline, a seasonal component that we modeled using a sine and cosine wave, and an offset given by the respective number of person years at risk. We excluded January to March, July, and August, and November and December in the estimation to reduce the effect of months that are particularly vulnerable to external factors such as winter flu epidemics or heat waves in the summer. This is in line with the procedure applied by EuroMOMO [19]. The model was fitted to the respective monthly death counts from 2015 to 2019. We used bootstrapping to address the uncertainty in observed death counts and to provide a prediction interval (PI) for the predicted mortality in 2020. We produced 5000 sets of resampled death counts, assuming that deaths are Poisson distributed with mean equal to the observed counts. The model was fitted separately to each of these sets. Across the resulting 5000 predictions for the months in 2020, we extracted the median estimate, as well as the estimates at 2.5% and 97.5% quantiles to calculate the 95% prediction interval (PI).

### 2.4 Excess mortality

Excess mortality was calculated by comparing the observed death counts and death rates in 2020 with the median and 95% prediction interval (PI) of the expected mortality in 2020 in the absence of COVID-19. We estimated both absolute levels of excess mortality, presented as death rates, and difference in death counts, and the relative mortality difference between the observed and expected mortality level within each care group and by sex and age.

Finally, to compare the estimated excess of mortality with the COVID-19 death toll, we used numbers of COVID-19 deaths by age group and care status, published as publicly available statistics by the National Board of Health and Welfare in Sweden. We estimated to what extent COVID-deaths account for the total excess in mortality during the months April-June in 2020 stratified by age group.

### 2.5 Role of the funding source

The funding agency had no role in study design, data collection, data analysis, interpretation or writing of the report.

## 3 Results

The proportion of the population, in men and women, respectively, who belong to each of the three groups at different ages can be found in Supplementary Fig. 1, the left panel showing the distribution during 2015–19 and the right panel, 2020. Before age 80, the proportion of individuals with public financed care, at own home or in care homes, did not exceed 5%. Compared to men, a higher proportion of women received care. In age group 85–89, around 25% of women received either home care or lived in a care home, while the corresponding proportion in men was around 20%. In age group 90+, more than 50% of the population received home care or lived in a care home. Even if subject to small variations, the distributions have been rather stable over the study period.

**Fig. 1:**
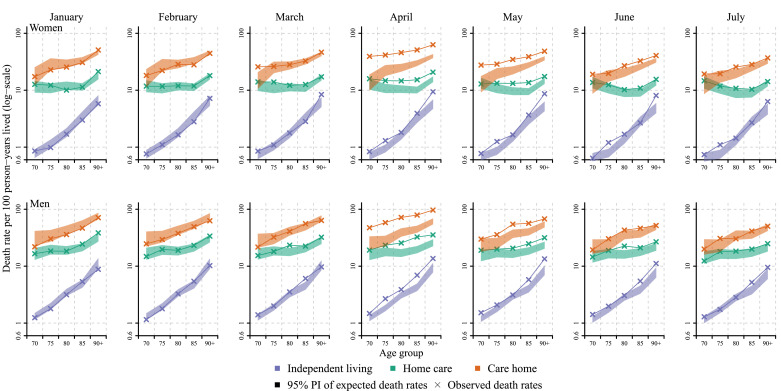
Age- and sex-specific observed death rates per month in 2020 compared to the 95% prediction interval of expected death rates, stratified by level of care, Sweden. Notes: Expected death rates (in the absence of COVID-19) have been predicted from a Poisson regression, with a time and a seasonal component, fitted for the months April to June and September to October between 2015 and 2019. The 95% prediction interval (shaded area) is based on 5000 predictions from the model using bootstrapped samples of the corresponding age-specific death counts.

Fig. 1 presents the observed and expected death rates by sex and over age from January to July 2020. The figure clearly shows pronounced mortality differences between the three groups regardless of the pandemic, with individuals in care homes having around ten to twenty times higher death rates compared to individuals in independent living at comparable ages. The differences were larger among the younger old than among the oldest old. The figure further shows that in the months prior to the pandemic, the observed death rates for 2020 were at the lower end of the prediction interval of the expected death rates. From April onwards, when the pandemic had a clear impact on death rates, excess mortality was primarily present in the two groups who received care, while the group in independent living showed little excess mortality. Finally, the figure shows that the COVID-19 pandemic seems to have shifted mortality to higher levels almost in parallel to the age trajectory we would expect without the pandemic. To wit, the excess mortality was not more present in the oldest old than in the younger old compared to each age groups’ expected levels of mortality, with some minor exceptions. For example, in May, the largest excess in the independent living group was experienced by those aged 90+. By July, almost all mortality levels were within the range of the expected mortality for 2020.

**Fig. 2:**
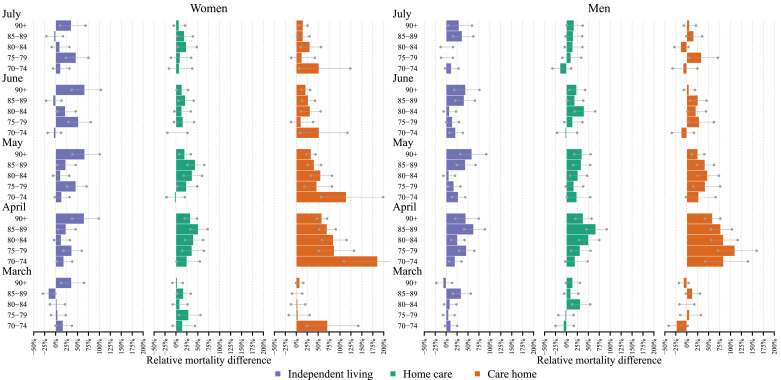
Relative differences in observed vs expected age- and sex-specific monthly death rates in 2020, stratified by level of care, March to July 2020, Sweden. Gray bars show the 95% prediction interval. Notes: Relative mortality differences have been calculated by diving death rates in 2020 by the corresponding predicted death rates minus one. Expected death rates have been predicted from a Poisson regression, with a time and a seasonal component, fitted for the months April to June and September to October between 2015 and 2019. The 95% prediction interval (gray bars) is based on the 5000 predictions from the model using bootstrapped samples of the corresponding age-specific death counts.

Fig. 2 shows the relative differences in excess mortality within each care group, and by sex and age. While the relative increase in excess mortality was largest in April and May for the two groups with care, the pattern was even more pronounced over these months for the group in independent living. In this group, it was particularly pronounced the oldest individuals – among women aged 90+ and men 85+ - who experienced consistently higher death rates than expected, corresponding to around 70% higher than expected for women and 50% higher for men from April to June. As seen in Fig. 1, the group living in care homes experienced the greatest increases in age-specific death rates, corresponding to 75% or more in ages 70–90 for men and ages 70–84 for women. Above that age, the excess mortality was around 50% for both men and women, lower than among the oldest age group in independent living.

**Fig. 3:**
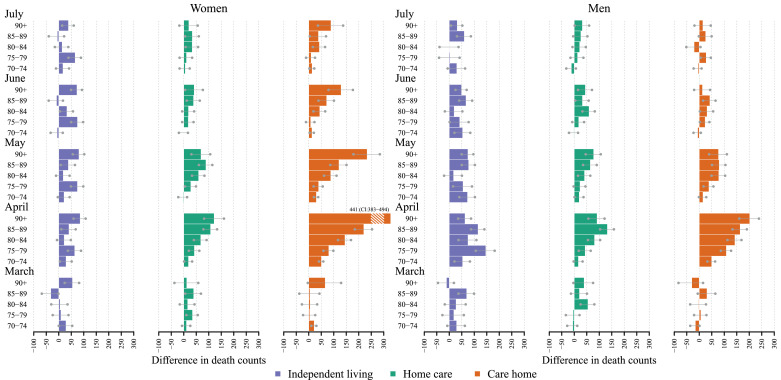
Absolute differences in observed vs expected age- and sex-specific monthly death counts in 2020 stratified by level of care, March to July 2020, Sweden. Gray bars show the 95% prediction interval. Notes: Differences have been calculated by subtracting death counts in 2020 by the corresponding expected death counts. Expected death counts have been estimated from a Poisson regression, with a time and a seasonal component, fitted for the months April to June and September to October between 2015 and 2019. The 95% prediction interval is based on the 5000 predictions from the model using bootstrapped samples of the corresponding age-specific death counts. The bars depict the difference with the median prediction and the dots refers to difference using the boundaries of 95% prediction interval.

However, even if the excess mortality was lower in a relative sense in the oldest age group in care homes, this group had the highest absolute number of excess deaths (Fig. 3). In fact, during April, the number of excess deaths was higher at every age in the care home group compared to the other two groups. Although Fig. 1 related the number of excess deaths to the size of each group, Fig. 3 presents only crude death counts, as illustrated by the rather low contribution of deaths in the youngest age group in care home settings. Even though this group had the highest relative excess mortality, it did not substantially impact overall excess mortality because so few individuals live in care homes at this age. At no time were there fewer deaths than expected for any of the age groups. The highest contribution of death counts was seen in April in women aged 90+ living in care homes, with 441 more deaths than expected (PI: 383–494). The corresponding number for men was 202 (PI: 160–239).

**Fig. 4:**
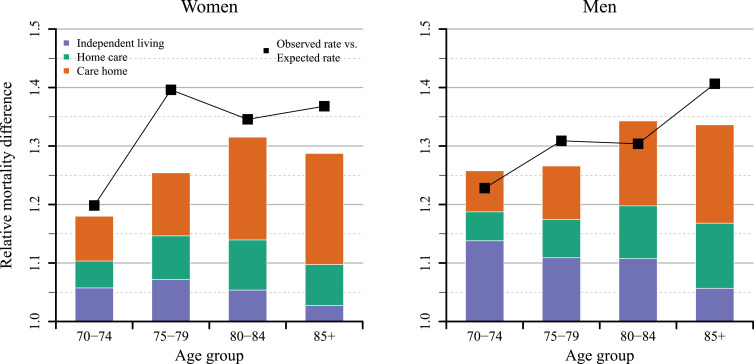
Relative differences between observed and expected age- and sex-specific death rates during April to June 2020 for Sweden, as well as the share that COVID-19 related deaths make up to the difference, by care status. Notes: The expected death rates for the total Swedish population has been calculated by adding all expected death counts by age and level of care and dividing these counts by the corresponding number of person years. The bars are based on death rates, where COVID-19 related deaths are added stepwise to the expected number of death counts. The black dots mark the difference between the observed death rate in 2020 and the prediction for the total population. Differences have been calculated by diving death rates in 2020 by the corresponding expected death rates minus one. Only the median of the prediction has been used. Expected death counts have been predicted from a Poisson regression with a time and a seasonal component, fitted for the months April to June and September to October between 2015 and 2019.

Fig. 4 presents two things, first, the total relative excess mortality for the entire period from April to June 2020 for the three groups, by age and sex (black line), as well as the share of those deaths attributable to COVID-19 deaths (bars). For example, among women aged 70–74, we observe a relative excess in death rate of around 5%. Additionally, adding in the respective COVID-19 deaths in women receiving home care, the relative excess in the death rate rises to 10%. Finally, also adding the deaths in women in care homes results in relative excess of 17.5%. The total relative excess observed for this age group was around 20%.

The relative excess mortality in the total population, during the whole period from April to June, in Sweden, was largest among women aged 75–79 years and men aged 85+, corresponding to around 40% higher than expected. COVID-19 related deaths accounted for most of the excess mortality; however, for women aged 75–79, there was a deviation. Fig. 4 further shows how individuals in care homes account for the largest share of the total excess mortality over the whole period at all ages, except men aged 75–79. This pattern was more pronounced among women than men. In women, most COVID-19 deaths came from individuals in care homes, particularly in the 85+ age group, compared to about half of the deaths in men. The share of excess mortality deaths among individuals in independent living declined with age. For the age group 85+, it was only 5% among women and 7% among men.

### 3.1 Stockholm

The results for the most affected region in Sweden, Stockholm, show the same overall pattern as for the rest of Sweden. However, excess mortality started earlier and was higher than in the rest of Sweden (Supplementary materials).

## 4 Discussion

To the best of our knowledge, this is the first study that estimated and compared excess mortality during the COVID-19 pandemic in population groups stratified by level of care (i.e., independent living, home care, and care home) and age. Most earlier studies have explored mortality differences by age and comorbidity based only on individuals infected by COVID-19. Based on these studies, it was unclear how the increase in mortality with age had been affected by the pandemic.

Our results showed that before the pandemic began, observed mortality levels were similar to, or even at the lower end of, the predicted death rates for 2020 in Sweden. In March 2020, excess mortality was observed only in young-old women residing in care homes, whereas in April there was excess mortality in all three groups and at all ages. However, excess mortality was more pronounced among those receiving care than among the group in independent living. The relative increase in mortality with age was not larger during 2020 than what was expected from previous years. For the group living in care homes, we instead observed higher excess mortality among the younger ages than among the oldest old. Because excess mortality is estimated as the difference between observed and expected mortality, the magnitude depends on the predicted level of the expected mortality. We chose a regression approach to model our baseline because we compared three population groups with varying degrees of mortality improvement. However, in sensitivity analyses related to model specification and data input, where we tested the inclusion and exclusion of different months, the shape of the seasonal component, and the inclusion of 2020 summer months, we found the age pattern for mortality was consistent (supplementary figures 6, 7). That is, despite varying magnitudes of excess mortality, the finding that the relative effect of age on mortality seems to be similar during the pandemic as before the pandemic. This is not to be confused with the fact that age is indeed a strong predictor of death, and that the level of mortality was clearly highest at higher ages.

Taken together, our results suggest that the comorbidity burden, measured by the level of care, plays an important role in the risk of COVID-19 associated death, regardless of age. This interpretation is supported by the gradient dose-response relationship between care and mortality, where care home residents experienced the greatest excess mortality, followed by those with home care, and finally those living independently. Furthermore, the results suggest that age alone is not necessarily a risk factor for COVID-19-specific death, beyond the “normal” risk of age that is present in absence of the pandemic. This interpretation is supported by the parallel shift in death rates over age observed in all three groups, rather than an exponential increase with age. This finding is in line with results of a recent US study [20], even if that study was restricted to individuals younger than 84 years.

It is likely that our findings also reflect the risk of exposure to the virus, in addition to the risk of dying from COVID-19. Although Individuals receiving care regularly meet home care providers, the group in independent living is likely to have fewer contacts outside their household. Even if the relatively healthy group in independent living may have been exposed to the virus during the early phase of the pandemic through social events and everyday living, they may have been able to avoid the virus to a higher extent by self-isolation. In Sweden, individuals 70 years and older were identified as a risk group and recommended to stay in isolation in late March 2020, something those with home care and in care homes could not do. Official statistics of confirmed COVID-19 in the groups with home care and in care homes in July 2020 (National Board of Health and Welfare) indeed show that the proportion of confirmed COVID-19 cases was higher in care homes than in home care. This may indicate that individuals in care homes were more affected, and possibly also tested to a greater extent. The differences in positive tests vary greatly between age groups. For instance, in July 2020, for men, it differs between 0.5 to 11 percentage points depending on age, and among women between 0 and 27 percentage points. Because we also observed an age pattern of excess mortality in age groups with no difference in positive tests, we conclude that even if a different level of exposure to the virus is a likely explanation, it may not account for all of the difference in mortality.

In line with previous studies, we found higher excess mortality among men than women during the pandemic, but the differences appear to be smaller when broken down by level of care. The finding that the sex ratio of COVID-19 mortality varies with age is consistent with past findings [21]. It must be noted, however, that observed mortality in 2020 is compared to predicted mortality based on the previous five-year trend, and, especially for women in independent living aged 75–79 and 90+, the improvement in the previous years was surprisingly strong (supplementary figure 6). As the expectation for improved mortality in 2020 continues this trend, the difference between expected and observed may be especially inflated. Though large improvements in recent years may be the result of fluctuations and could be followed by a decline. Therefore, the excess mortality in these two groups may be somewhat overestimated.

The considerably higher number of absolute excess deaths among women living in care homes is likely driven by the higher number of women living in care homes. We found higher excess mortality among males in independent living compared to women, possibly reflecting the fact that men of the same health status as women are less likely to receive home care because of support provided by their wives. In Sweden, it is more common for men than women to receive informal care [22]. Our data also show that fewer men than women of the same age received care, indicating that level of care may represent a better health proxy for women than men.

The observed difference between all-cause mortality and COVID-19 deaths, primarily among women ages 75–79, can be explained by several factors. For instance, it may be that excess deaths are due to other causes besides COVID-19, that deaths were in reality caused by COVID-19 but individuals were not tested, or that the estimation of the expected deaths for 2020 based on previous years was too optimistic.

It has been suggested that part of the excess mortality during the first wave of the pandemic reflects deaths occurring somewhat earlier than they otherwise would have among the frailest. If this were true, one would have expected a more pronounced reduction in mortality in the care home group compared to the group in independent living in July. We did not see this, at least not yet.

The strengths of the present study include its population-based setting with data available on both deaths and the corresponding population at risk, reliable register information regarding living arrangement and deaths, and the possibility to use level of care as a proxy for health status. Additionally, our use of a regression approach to estimate baseline mortality to account for different levels of mortality improvement in the past, and therefore avoid expected mortality levels to be too pessimistic for groups with improvement and ensured that mortality was not underestimated.

Some limitations of our study need mentioning. First, based on the available data it was not possible to identify the mechanism behind the relatively higher excess mortality in the two groups with care compared to the group in independent living. This could reflect a higher death risk due to poorer health status and a higher comorbidity burden, but differences in exposure to the virus depending on frequency of contact with health providers may also be at play. Also, a potential drawback of using excess deaths is that they mirror a net effect of both excess deaths and potentially saved lives during the pandemic. However, among individuals aged 70 and older, the pandemic is unlikely to have had any effect on saving lives, even if the seasonal flu was milder than most winters.

## 5 Conclusion

We found stepwise elevated excess mortality by level of care during the first wave of the COVID-19 pandemic in Sweden. We also found that mortality increased with age but that the relative effect of age on mortality is no different during the pandemic than otherwise. Of special note was the relatively higher excess mortality among groups receiving care, suggesting that health status plays a more important role than age for COVID-19 associated deaths. Part of our findings may be attributed to differences in exposure to the virus between individuals receiving formal care and those living independently.

## Author Contributions

KM jointly conceived the study with ME, AA and ML. KM provided the data and ME analysed the data and created the figures. KM and ME have both verified the underlying data. KM and ME wrote the manuscript and AA and ML edited the manuscript. KM coordinated the project. All authors read and approved the final version of the manuscript.

## Data sharing

Aggregated data and codes for the estimation can be made available by the authors. Enquiries should be made to the corresponding author.

Ethics committee approval was not required since the study was based on aggregated data.

## Declaration of Interests

None of the authors have anything to disclose.

## Appendix Supplementary materials

Image, application 1

Image, application 2

Image, application 3

## References

[1] Shahid Z., Kalayanamitra R., McClafferty B., Kepko D., Ramgobin D., Patel R., et al. COVID-19 and older adults: what we know. 2020;68(5):926–9. 10.1111/jgs.16472 PMC7262251 32255507

[2] Yang J., Zheng Y., Gou X., Pu K., Chen Z., Guo Q. (2020). Prevalence of comorbidities and its effects in patients infected with SARS-CoV-2: a systematic review and meta-analysis. Int J Infect Dis.

[3] Montero-Odasso M., Hogan D.B., Lam R., Madden K., MacKnight C., Molnar F. (2020). Age alone is not adequate to determine health-care resource allocation during the COVID-19 pandemic. Can Geriatr J.

[4] Romero Starke K., Petereit-Haack G., Schubert M., Kämpf D., Schliebner A., Hegewald J. (2020). The age-related risk of severe outcomes due to COVID-19 infection: a rapid review, meta-analysis, and meta-regression. Int J Environ Res Public Health.

[5] Hägg S., Jylhävä J., Wang Y., Xu H., Metzner C., Annetorp M. (2020). Age, frailty and comorbidity as prognostic factors for short-term outcomes in patients with COVID-19 in geriatric care. J Am Med Dir Assoc.

[6] Harrison S.L., Fazio-Eynullayeva E., Lane D.A., Underhill P., Lip G.Y.H. (2020). Comorbidities associated with mortality in 31,461 adults with COVID-19 in the United States: a federated electronic medical record analysis. PLoS Med.

[7] Vaupel J.W (2010). Biodemography of human ageing. Nature.

[8] Palaiodimos L., Kokkinidis D.G., Li W., Karamanis D., Ognibene J., Arora S. (2020). Severe obesity, increasing age and male sex are independently associated with worse in-hospital outcomes, and higher in-hospital mortality, in a cohort of patients with COVID-19 in the Bronx, New York. Metabolism.

[9] Li X., Xu S., Yu M., Wang K., Tao Y., Zhou Y. (2020). Risk factors for severity and mortality in adult COVID-19 inpatients in Wuhan. J Allergy Clin Immunol.

[10] Aburto J.M., Kashyap R., Scholey J., Angus C., Ermisch J., Mills M. (2020). Estimating the burden of COVID-19 on mortality, life expectancy and lifespan inequality in England and Wales: a population-level study. medRxiv.

[11] Kontis V., Bennett J.E., Rashid T., Parks R.M., Pearson-Stuttard J., Guillot M. (2020). Magnitude, demographics and dynamics of the effect of the first wave of the COVID-19 pandemic on all-cause mortality in 21 industrialized countries. Nat Med.

[12] Vestergaard L.S., Nielsen J., Richter L., Schmid D., Bustos N., Braeye T. (2020). Excess all-cause mortality during the COVID-19 pandemic in Europe - preliminary pooled estimates from the EuroMOMO network, March to April 2020. Euro Surveill.

[13] Hollinghurst J., Lyons J., Fry R., Akbari A., Gravenor M., Watkins A. (2021). The impact of COVID-19 on adjusted mortality risk in care homes for older adults in Wales, UK: a retrospective population-based cohort study for mortality in 2016-2020. Age Ageing.

[14] Schön P., Lagergren M., Kåreholt I. Rapid decrease in length of stay in institutional care for older people in Sweden between 2006 and 2012: results from a population-based study. 2016;24(5):631–8. 10.1111/hsc.12237 25944315

[15] Sveriges Kommuner och Landsting. Fakta om äldreomsorgen i ljuset av corona-pandemin.; 2020. in English: facts about the elderly care during the Covid-19 pandemic. https://rapporter.skr.se/fakta-om-aldreomsorgen.html

[16] Brooke H.L., Talback M., Hornblad J., Johansson L.A., Ludvigsson J.F., Druid H. (2017). The Swedish cause of death register. Eur J Epidemiol.

[17] Dudel C., Riffe T., Acosta E., van Raalte A., Strozza C., Myrskylä M. (2020). Monitoring trends and differences in COVID-19 case-fatality rates using decomposition methods: contributions of age structure and age-specific fatality. PLoS ONE.

[18] Serfling R.E. (1963). Methods for current statistical analysis of excess pneumonia-influenza deaths. Public Health Rep.

[19] Gergonne B., Mazick A., O'Donnell J., Oza A., Cox B., Wuillaume F., Andrews N. (2011).

[20] Sasson I. Aging and COVID-19 mortality: a demographic perspective. 2020

[21] Bhopal S.S., Bhopal R. (2020). Sex differential in COVID-19 mortality varies markedly by age. Lancet.

[22] Dahlberg L., Berndt H., Lennartsson C., Schön P. (2018). Receipt of formal and informal help with specific care tasks among older people living in their own home. Natl Trends Over Two Decades.

