# Supplementary material for: Excess mortality for men and women above age 70 according to level of care during the first wave of COVID-19 pandemic in Sweden: A population-based study
Source: Lancet Reg Health Eur. 2021 Mar 17;4:100072. doi: 10.1016/j.lanepe.2021.100072 (PMC8454796; doi:10.1016/j.lanepe.2021.100072)
Supplement: Supplementary file 1 [file mmc1.docx]

STROBE Statement—Checklist of items that should be included in reports of ***cohort studies***

|  | Item No | Recommendation |
| --- | --- | --- |
| **Title and abstract** | 1 | (*a*) Indicate the study’s design with a commonly used term in the title or the abstract **Yes, we refer to estimation of excess mortality in 2020 based on comparing to previous year using Poisson regression.** |
|  |  | (*b*) Provide in the abstract an informative and balanced summary of what was done and what was found. **Yes** |
| Introduction | | |
| Background/rationale | 2 | Explain the scientific background and rationale for the investigation being reported. **Yes** |
| Objectives | 3 | State specific objectives, including any prespecified hypotheses. **Yes** |
| Methods | | |
| Study design | 4 | Present key elements of study design early in the paper. **Yes** |
| Setting | 5 | Describe the setting, locations, and relevant dates, including periods of recruitment, exposure, follow-up, and data collection. **Yes** |
| Participants | 6 | (*a*) Give the eligibility criteria, and the sources and methods of selection of participants. Describe methods of follow-up. **Yes, however full national coverage in registers.** |
|  |  | (*b*) For matched studies, give matching criteria and number of exposed and unexposed. **Not relevant.** |
| Variables | 7 | Clearly define all outcomes, exposures, predictors, potential confounders, and effect modifiers. Give diagnostic criteria, if applicable. **Exposure here is the level of care for which all analyses are stratified for.** |
| Data sources/ measurement | 8* | For each variable of interest, give sources of data and details of methods of assessment (measurement). Describe comparability of assessment methods if there is more than one group. **Yes.** |
| Bias | 9 | Describe any efforts to address potential sources of bias. **Yes, we performed several sensitivity analyses in order to test the robustness of the expected mortality levels. In addition we discuss limitations and their potential consequences in the manuscript.** |
| Study size | 10 | Explain how the study size was arrived at. **Full national coverage, no sample.** |
| Quantitative variables | 11 | Explain how quantitative variables were handled in the analyses. If applicable, describe which groupings were chosen and why. **Yes.** |
| Statistical methods | 12 | (*a*) Describe all statistical methods, including those used to control for confounding. **Yes, even if confounding is not relevant since we do not estimate any causal pathways but present descriptive epidemiology.** |
|  |  | (*b*) Describe any methods used to examine subgroups and interactions. **Subgroups are stratified for, interaction was not tested.** |
|  |  | (*c*) Explain how missing data were addressed. **No missing data.** |
|  |  | (*d*) If applicable, explain how loss to follow-up was addressed. **No loss to follow up.** |
|  |  | (*e*) Describe any sensitivity analyses. **Yes, this is done in the manuscript.** |
| Results | | |
| Participants | 13* | (a) Report numbers of individuals at each stage of study—eg numbers potentially eligible, examined for eligibility, confirmed eligible, included in the study, completing follow-up, and analysed. **We present distribution of the population at different ages and years. Since we are looking at the total population in three groups exact numbers are not given.** |
|  |  | (b) Give reasons for non-participation at each stage. **Not relevant here.** |
|  |  | (c) Consider use of a flow diagram. **Not relevant here.** |
| Descriptive data | 14* | (a) Give characteristics of study participants (eg demographic, clinical, social) and information on exposures and potential confounders. **Not relevant beyond level of care, age and sex for the purpose of this study.** |
|  |  | (b) Indicate number of participants with missing data for each variable of interest. **No missing data.** |
|  |  | (c) Summarise follow-up time (eg, average and total amount). **All follow up are based on exact person time from register data and presented as rates in the manuscript.** |
| Outcome data | 15* | Report numbers of outcome events or summary measures over time. **Yes, we present number of deaths, and death rates.** |
| Main results | 16 | (*a*) Give unadjusted estimates and, if applicable, confounder-adjusted estimates and their precision (eg, 95% confidence interval). Make clear which confounders were adjusted for and why they were included. **Not relevant here.** |
|  |  | (*b*) Report category boundaries when continuous variables were categorized. **Not relevant here.** |
|  |  | (*c*) If relevant, consider translating estimates of relative risk into absolute risk for a meaningful time period. **Yes, we present both absolute and relative estimates.** |
| Other analyses | 17 | Report other analyses done—eg analyses of subgroups and interactions, and sensitivity analyses. **Yes we present several additional results in supplementary materials.** |
| Discussion | | |
| Key results | 18 | Summarise key results with reference to study objectives. **Yes.** |
| Limitations | 19 | Discuss limitations of the study, taking into account sources of potential bias or imprecision. Discuss both direction and magnitude of any potential bias. **Yes this is done in the discussion, given the allowed space of elaboration.** |
| Interpretation | 20 | Give a cautious overall interpretation of results considering objectives, limitations, multiplicity of analyses, results from similar studies, and other relevant evidence. **Yes**. |
| Generalisability | 21 | Discuss the generalisability (external validity) of the study results. **This is done to some extent but cannot be generalized since it does not refer to etiological association but descriptive data of the pandemics effect in Sweden.** |
| Other information | | |
| Funding | 22 | Give the source of funding and the role of the funders for the present study and, if applicable, for the original study on which the present article is based. **Yes.** |

*Give information separately for exposed and unexposed groups.

**Note:** An Explanation and Elaboration article discusses each checklist item and gives methodological background and published examples of transparent reporting. The STROBE checklist is best used in conjunction with this article (freely available on the Web sites of PLoS Medicine at http://www.plosmedicine.org/, Annals of Internal Medicine at http://www.annals.org/, and Epidemiology at http://www.epidem.com/). Information on the STROBE Initiative is available at http://www.strobe-statement.org.
