# Supplementary material for: Excess mortality for men and women above age 70 according to level of care during the first wave of COVID-19 pandemic in Sweden: A population-based study
Source: Lancet Reg Health Eur. 2021 Mar 17;4:100072. doi: 10.1016/j.lanepe.2021.100072 (PMC8454796; doi:10.1016/j.lanepe.2021.100072)
Supplement: Supplementary file 2 [file mmc2.pdf]

Supplemental materials: Excess mortality for men and women above age 70 living in care homes, with home care and in independent living during the first wave of the COVID-19 pandemic in Sweden

Karin Modig* Mats Lambe† Anders Ahlbom* Marcus Ebeling‡

January 26, 2021

*Karolinska Institutet, Stockholm, Sweden

†Karolinska Institutet, Stockholm, Sweden & Regional Cancer Centre, University Hospital, Uppsala, Sweden

‡Max Planck Institute for Demographic Research, Rostock, Germany

1 Distribution of population by care status

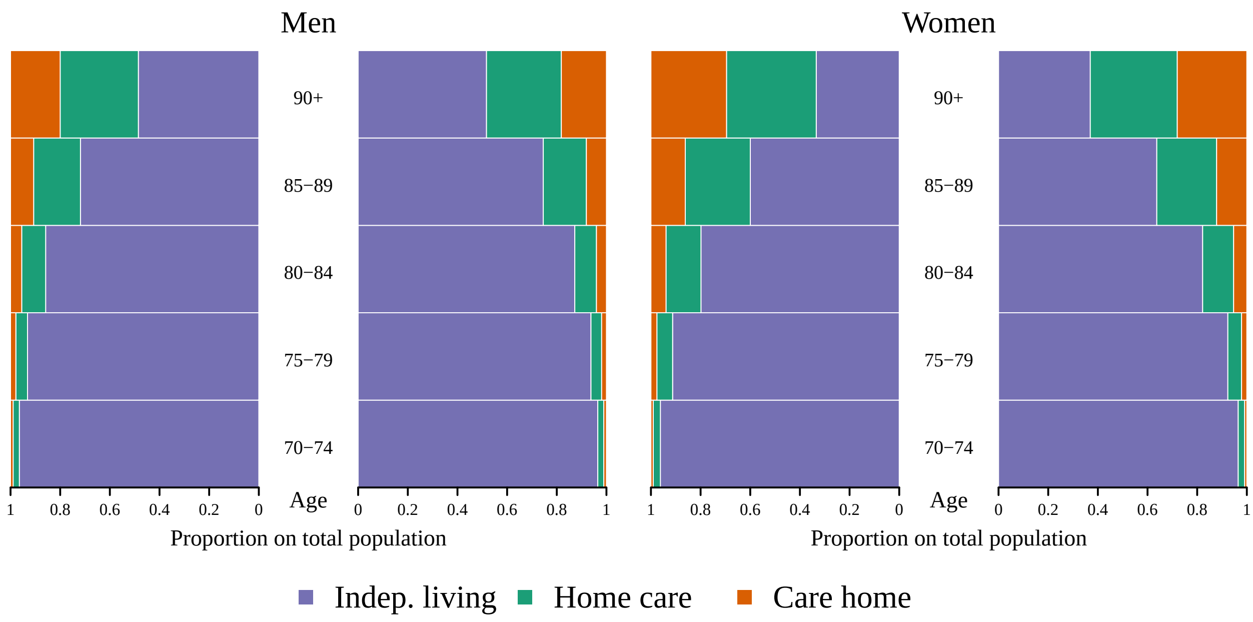

Figure 1: Age-specific proportions of the care-specific subpopulations on the total population separately averaged across months for the periods January 2015 to December 2019 and January 2020 to August 2020, women and men, Sweden.

2 Estimates for Stockholm county

2.1 Expected mortality and sensitivity analysis

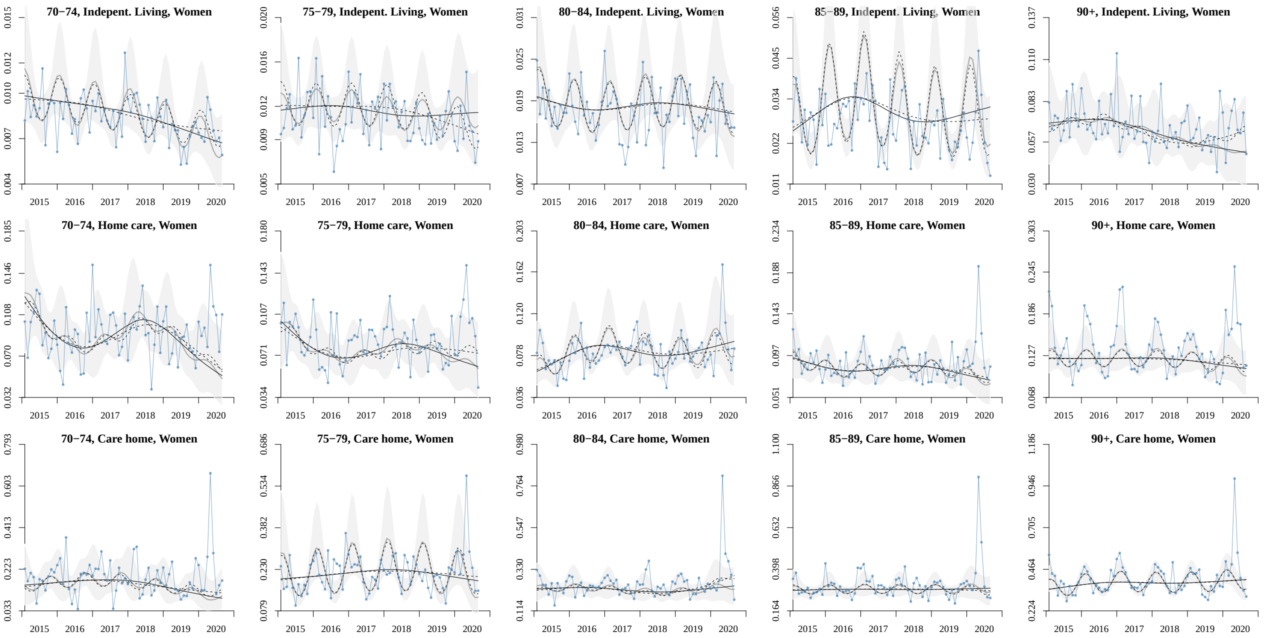

Figure 2: Observed death rates (blue circles), median of expected death rates (gray line), 95% prediction interval of expected death rates (shaded area), time trend of the median expected death rates (black solid line), median of expected death rates including July 2020 in the model fit (dashed line) and time trend of the median expected death rates including July 2020 in the model fit (straight dashed line) by level of care and age group, women, 2015-2020, Stockholm county. Notes: Expected death rates have been predicted from a Poisson regression with a time and a seasonal component, fitted for the months April to June and September to October between 2015 and 2019. The 95% prediction interval is based on 5000 predictions from this model using bootstrapped samples of the corresponding age-specific death counts. To constrain the mortality change in 2020 and to evaluate the sensitivity of our results to expected mortality in 2020, we also estimated a model that includes, in addition to the previous setting, July 2020 in the parameter estimation.

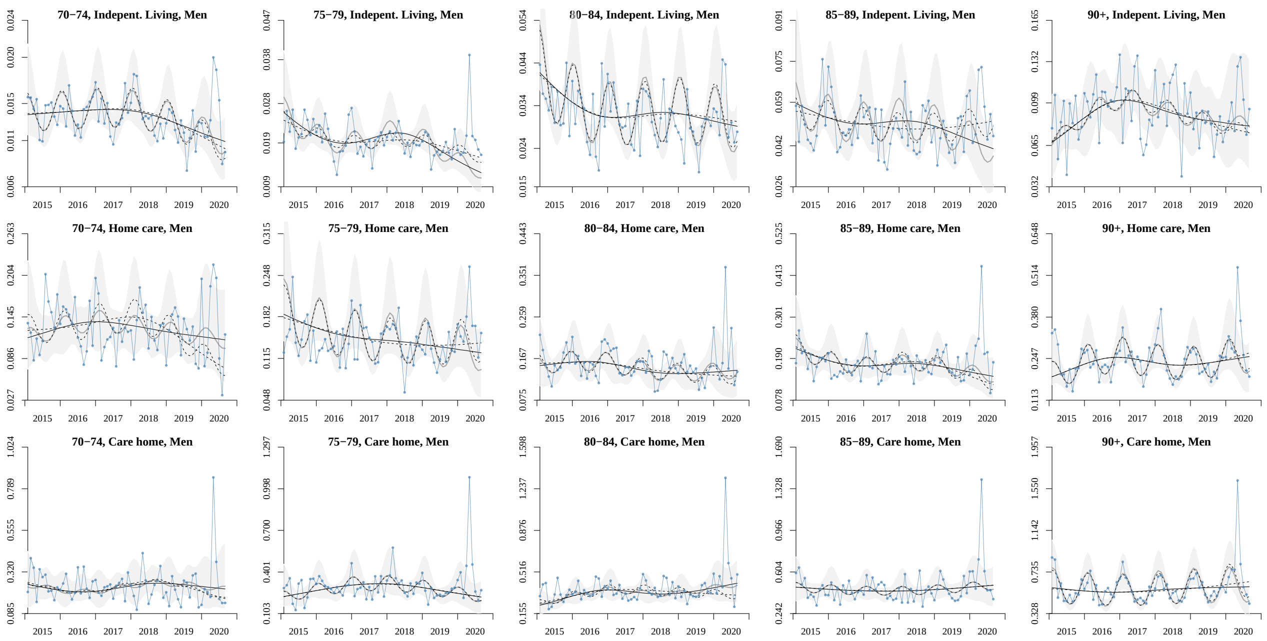

Figure 3: Observed death rates (blue circles), median of expected death rates (gray line), 95% prediction interval of expected death rates (shaded area), time trend of the median expected death rates (black solid line), median of expected death rates including July 2020 in the model fit (dashed line) and time trend of the median expected death rates including July 2020 in the model fit (straight dashed line) by level of care and age group, men, 2015-2020, Stockholm county. Notes: Expected death rates have been predicted from a Poisson regression with a time and a seasonal component, fitted for the months April to June and September to October between 2015 and 2019. The 95% prediction interval is based on 5000 predictions from the model using bootstrapped samples of the corresponding age-specific death counts. To constrain the mortality change in 2020 and to evaluate the sensitivity of our results to expected mortality in 2020, we also estimated a model that includes, in addition to the previous setting, July 2020 in the parameter estimation.

2.2 Observed vs expected death rates by level of care

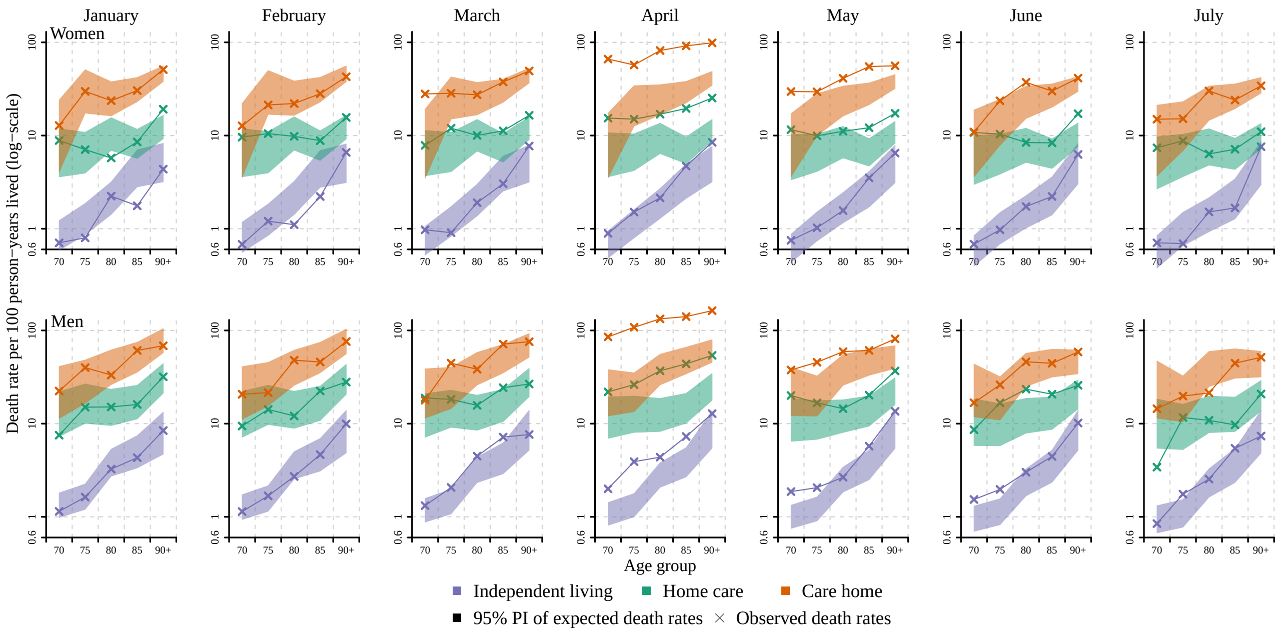

Figure 4: Age- and sex-specific observed death rates per month in 2020 compared to the 95county. Notes: Expected death rates (in the absence of Covid-19) have been predicted from a Poisson regression with a time and a seasonal component, fitted for the months April to June and September to October between 2015 and 2019. The 95% prediction interval (shaded area) is based on 5000 predictions from the model using bootstrapped samples of the corresponding age-specific death counts.

2.3 Excess deaths

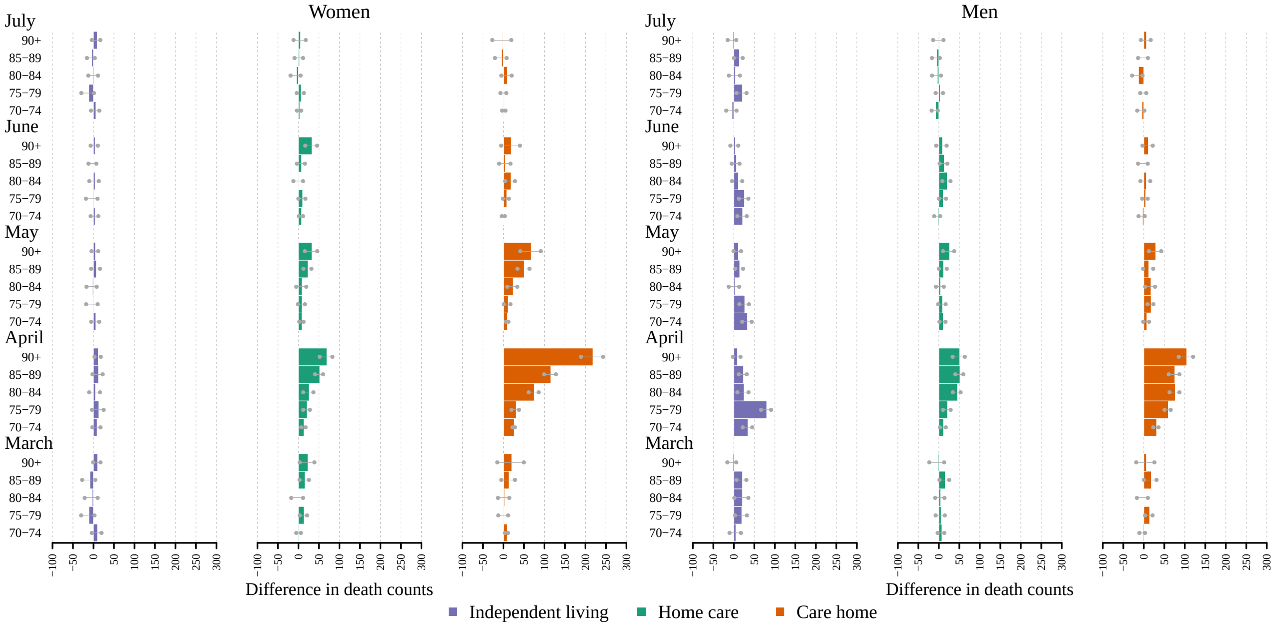

Figure 5: Absolute differences in observed vs expected age- and sex-specific monthly death counts in 2020 stratified by level of care, women and men, March to July 2020, Stockholm county. Notes: Differences have been calculated by subtracting the number of expected deaths from the death counts in 2020. Expected death counts have been predicted from a Poisson regression with a time and a seasonal component, fitted for the months April to June and September to October between 2015 and 2019. The 95% prediction interval is based on 5000 predictions from the model using bootstrapped samples of the corresponding age-specific death counts. The bars depict the difference with the median prediction and the dots refers to difference using the boundaries of 95% prediction interval.

3 Estimates for Sweden

3.1 Expected mortality and sensitivity analysis

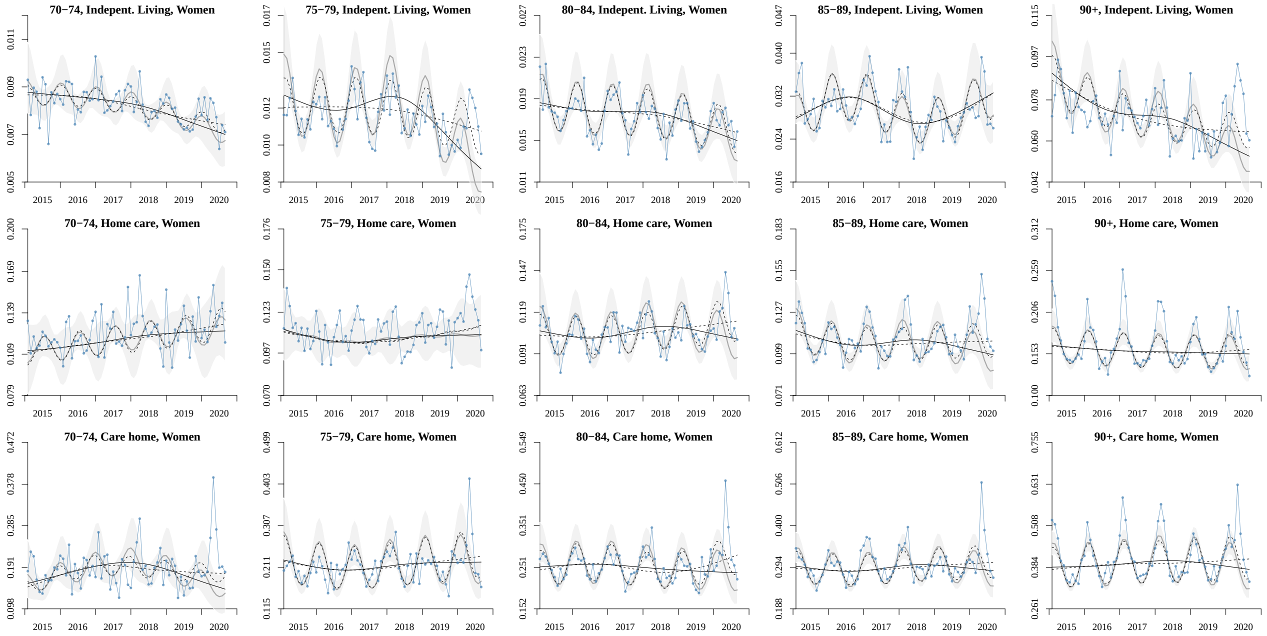

Figure 6: Observed death rates (blue circles), median of expected death rates (gray line), 95% prediction interval of expected death rates (shaded area), time trend of the median expected death rates (black solid line), median of expected death rates including July 2020 in the model fit (dashed line) and time trend of the median expected death rates including July 2020 in the model fit (straight dashed line) by level of care and age group, women, 2015-2020, Sweden. Notes: Expected death rates have been predicted from a Poisson regression with a time and a seasonal component, fitted for the months April to June and September to October between 2015 and 2019. The 95% prediction interval is based on 5000 predictions from the model using bootstrapped samples of the corresponding age-specific death counts. To constrain the mortality change in 2020 and to evaluate the sensitivity of our results to expected mortality in 2020, we also estimated a model that includes, in addition to the previous setting, July 2020 in the parameter estimation.

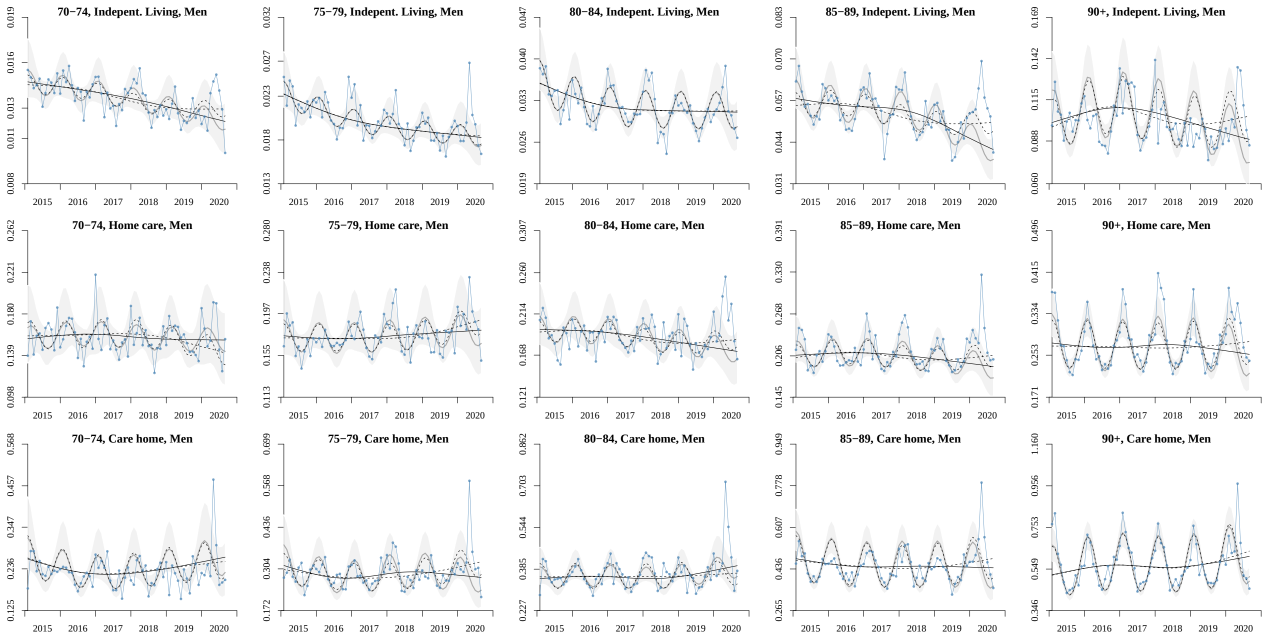

Figure 7: Observed death rates (blue circles), median of expected death rates (gray line), 95% prediction interval of expected death rates (shaded area), time trend of the median expected death rates (black solid line), median of expected death rates including July 2020 in the model fit (dashed line) and time trend of the median expected death rates including July 2020 in the model fit (straight dashed line) by level of care and age group, men, 2015-2020, Sweden. Notes: Expected death rates have been predicted from a Poisson regression with a time and a seasonal component, fitted for the months April to June and September to October between 2015 and 2019. The 95% prediction interval is based on 5000 predictions from the model using bootstrapped samples of the corresponding age-specific death counts. To constrain the mortality change in 2020 and to evaluate the sensitivity of our results to expected mortality in 2020, we also estimated a model that includes, in addition to the previous setting, July 2020 in the parameter estimation.

3.2 Sensitivity analysis of excess in death rates and death counts

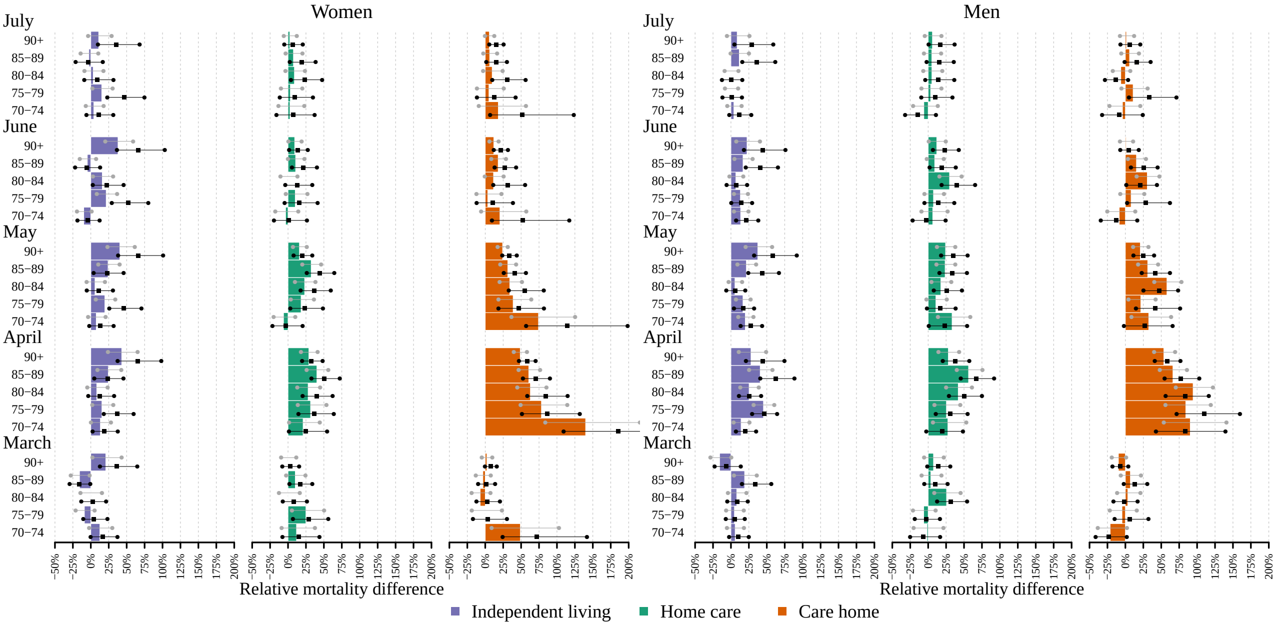

Figure 8: Relative differences in observed vs expected age- and sex-specific monthly death rates in 2020, stratified by level of care, women and men, March to July 2020, Sweden. Notes: Death rates in 2020 are compared to the median of expected death rates and the corresponding 95% prediction interval for the corresponding months based on a model fit including July 2020 (bars and gray dots) and the original model (excluding July, black dots and square). Relative mortality differences have been calculated by diving death rates in 2020 by the corresponding expected death rates minus one. Expected death rates have been predicted from a Poisson regression with a time and a seasonal component, fitted for the months April to June and September to October between 2015 and 2019. The 95% prediction interval is based on 5000 predictions from the model using bootstrapped samples of the corresponding age-specific death counts. To constrain the mortality change in 2020 and to evaluate the sensitivity of our results to expected mortality in 2020, we also estimated a model that includes, in addition to the previous setting, July 2020 in the parameter estimation. The black squares and dots refer to the median and the 95% prediction interval of the original model, and thus represent the estimates presented in the paper.

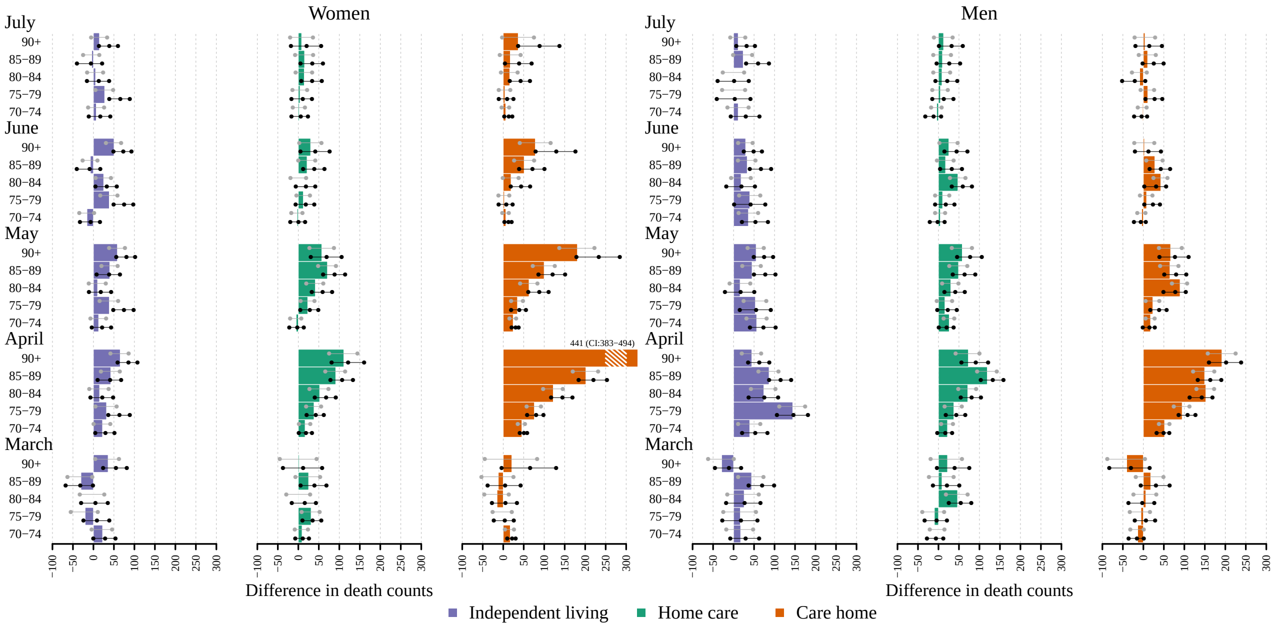

Figure 9: Absolute differences in observed vs expected age- and sex-specific monthly death counts in 2020 stratified by level of care, women and men, March to July 2020, Sweden. Notes: Death counts in 2020 are compared to the median of expected death counts and the corresponding 95% prediction interval for the corresponding months based on a model fit including July 2020 (bars and gray dots) and the original model (excluding July, black dots and square). Differences have been calculated by subtracting the number of expected deaths from the death counts in 2020. Expected death counts have been predicted from a Poisson regression with a time and a seasonal component, fitted for the months April to June and September to October between 2015 and 2019. The 95% prediction interval is based on 5000 predictions from the model using bootstrapped samples of the corresponding age-specific death counts. To constrain the mortality change in 2020 and to evaluate the sensitivity of our results to the expected mortality in 2020, we also estimated a model that includes, in addition to the previous setting, July 2020 in the parameter estimation. The black squares and dots refer to the median and the 95% prediction interval of the original model, and thus represent the estimates presented in the paper.
