# Supplementary material for: Excess mortality for men and women above age 70 according to level of care during the first wave of COVID-19 pandemic in Sweden: A population-based study
Source: Lancet Reg Health Eur. 2021 Mar 17;4:100072. doi: 10.1016/j.lanepe.2021.100072 (PMC8454796; doi:10.1016/j.lanepe.2021.100072)
Supplement: Supplementary file 3 [file mmc3.pdf]

I agree to be included in the Acknowledgements of this article

“Excess mortality for men and women above age 70 according to level of care during the first wave of COVID-19 pandemic in Sweden: a population-based study”

Enrique Acosta
Rostock 02/18/2021
